# Genetic Mutation Analysis in Small Cell Lung Cancer by a Novel NGS-Based Targeted Resequencing Gene Panel and Relation with Clinical Features

**DOI:** 10.1155/2021/3609028

**Published:** 2021-04-05

**Authors:** Wang Jin, Zhao Lei, Sun Xu, Zhou Fachen, Zhang Yixiang, Zhao Shilei, Guo Tao, Sun Zhe, Li Fengzhou, Wen-Hui Su, Gu Chundong

**Affiliations:** ^1^Department of Thoracic Surgery, The First Affiliated Hospital of Dalian Medical University, Dalian, Liaoning 116011, China; ^2^Department of Pathology, The First Affiliated Hospital of Dalian Medical University, Dalian, Liaoning 116011, China; ^3^Department of Biomedical Sciences, Graduate Institute of Biomedical Sciences, College of Medicine, Chang Gung Molecular Medicine Research Center, Chang Gung University, Taiwan

## Abstract

**Background:**

Small cell lung cancer (SCLC) is an aggressive and invasive malignancy that presents at advanced clinical stage with no more effective treatments. Development of a method for its early detection would be useful, also new therapeutic target need to be discovered; however, there is a lack of information about its oncogenic driver gene mutations.

**Objectives:**

We aim to identify the SCLC-related genomic variants that associate with clinical staging and serum protein biomarkers observed in other types of lung cancer.

**Methods:**

We screened formalin-fixed paraffin-embedded (FFPE) biopsy tissues of 32 Chinese SCLC patients using the 303 oncogenic driver gene panel generated by Tiling PCR amplification sequencing (tPAS) and analyzed the patients' corresponding serum protein levels of CYFRA21-1 CEA, NSE, and SCCA.

**Results:**

In total, we found 147 SCLC-related mutant genes, among these, three important genes (*TP53*, *RB1*, *KMT2D*) as well as five novel genes *LRRK2*, *BRCA1*, *PTCH1*, *ARID2*, and *APC* that altogether occurred in 90% of patients. Furthermore, increased mutations to 6 genes (*WT1*, *NOTCH1*, *EPHA3*, *KDM6A*, *SETD2*, *ACVR1B*) significantly associated with higher serum NSE levels (*P* = 0.0016) and higher clinical stages II + III compared to stage I (*P* = 0.06).

**Conclusions:**

Our panel is relatively reliable in detecting the oncogenic mutations of Chinese SCLC patients. Based on our findings, it may be possible to combine SCLC-related mutations and serum NSE for a simple detection of clinical staging.

## 1. Background

Lung cancer is a major cause of cancer-related deaths worldwide [1, 2]. Small cell lung cancer (*SCLC*), accounting for approximately 15% of lung cancers [3], is an aggressive, neuro-endocrine tumor characterized by a short doubling time, high growth rate, and early onset of metastases [4, 5]. While treatment includes surgery, chemotherapy and radiotherapy [6], the majority of patients present at advanced stages with loco-regional and systemic involvement and have poor prognosis and limited treatment options. Thus, a method for the early detection of SCLC is required and new treatments' targets need to be discovered.

There are no specific protein biomarkers identified for the SCLC. However, there are growing reports of a number of potentially useful serum proteins for the diagnosis of lung, prostate, and colorectal cancer [7–9]. Significantly, shorter survival rates have been associated with high levels of circulating tumor biomarkers, e.g., cytokeratin-19 fragment, neuron-specific enolase (NSE), and thymidine kinase, in patients with non-small-cell lung carcinoma (NSCLC) [10]. Tumor biomarkers can be further combined with certain mutations to improve the sensitivity for the detection of cancers, such as the carcinoembryonic antigen (CEA), carbohydrate antigen 19-9 (CA19-9), osteopontin, and hepatocyte growth factor that are combined with *KRAS* mutation to detect pancreatic cancer [11].

Due to the low incidence and survival rates from SCLC [12], as well as difficulty in obtaining sufficient tissue samples for research [13], still not enough is known about the driver mutations that associate with cancer development and progression. So far, exome sequencing of SCLC has highlighted the important mutations in the *TP53*, *RB1*, and histone-modifying genes [14]. Inactivating mutations in *TP53* and *RB1* are found in 65-90% of SCLC patients [14–17]. Truncating frameshift, splice, and nonsense site variants in the *KMT2D*, which encodes a histone H3K4 methyltransferase, are found in 8% of SCLC tumors and 17% of SCLC cell lines [18].

To increase our understanding of the mutational signatures of SCLC and assist with development of new diagnostic tools, we analyzed the formalin fixed and paraffin embedded (FFPE) biopsy specimens of 32 Chinese SCLC patients with a new technology called Tiling PCR amplification sequencing (tPAS) based on the sequencing of multiplex PCR amplicons, which can simultaneously genotype single nucleotide variants (SNVs), insertions or deletions (indels), and copy number variations (CNVs) in a panel of 303 cancer genes, selected from the Catalogue of Somatic Mutations in Cancer [19], Cancer Genome Atlas [20, 21], and Oncomine database [22].

## 2. Materials and Methods

### 2.1. Study Participants

We recruited 32 patients diagnosed with SCLC into this study. The tumor specimens were obtained from patients by surgeons in the Oncology Department of The First Affiliated Hospital of Dalian Medical University. All participants gave written informed consent for the provision of clinical information, biospecimen collection, and analysis. This study was approved by the Dalian Medical University and Hospital Research Ethics Committee. The Institutional Review Board (IRB) for this study is YJ-JG-QX-2018-146.

### 2.2. Pathological Analysis of Tumor Specimens

Tumor specimens were acquired by surgery (>2% of total tissue mass and >150 cells). Diagnosis of SCLC was confirmed by pathologists using histologic evaluation of the stained FFPE tumor sections. TNM staging system of International Association for the Study of Lung Cancer (version 7) was used to determine the clinical staging.

### 2.3. Extraction of DNA from Formalin Fixed Paraffin Embedded (FFPE) Biopsy Specimens

Macrodissection was performed to increase the tumor tissue percentage to ~80% prior to DNA extraction. Five to ten FFPE sections (5 mm thickness) of each tumor specimen were used to prepare the genomic DNA for mutation profiling. DNA was extracted from FFPE tissue using the AmoyDxò FPPE DNA Kit and DNA purification spin columns (Amoy, Xiamen, Fujian). All purified DNA samples were determined to be of high quality by spectroscopy analysis and were suitable for mutation analysis by the tPAS assay.

### 2.4. Targeted Next-Generation Sequencing (NGS)

Targeted NGS was performed on the 32 FFPE samples. Libraries were prepared according to Paragon Genomics manufacturer's protocol. Briefly, 40 ng of human DNA was used for each multiplex PCR reaction. CleanPlex™ panels were supplied with 2x concentrated primer pools. Using thin-wall PCR strip tubes, components were added to run the PCR program for amplify target DNA regions. For panels consisting of multiple primer pools, 10 *μ*l multiplex PCR reactions were combined for each sample. Magnetic bead suspensions were vortexed vigorously, and 1.3x sample volume of magnetic bead suspension was added to each sample. After beads were drawn onto one side of the wall, the supernatant was removed and discarded without touching the beads, 10 *μ*l TE buffer was added to each tube, and then, the DNA was immediately released from the beads. Amplicons were purified, and concentration of the library was measured.

### 2.5. Tiling PCR Amplification Sequencing (tPAS)

Paired-end 2 × 150 base pairs (bp) reads were generated from the Amplicon libraries using NextSeq CN500. Adaptor and low-quality bases in raw reads were trimmed using flexbar version v2.5 and aligned to the human reference genome hg19 using BWA-MEM version 0.7.5a. GATK v3.6 was used for local indel realignment and base quality recalibration [23]. GATK MuTect2 was used in somatic mutation (single nucleotide variants and small insertions and deletions) calling [24]. Somatic variant loci were defined by loci that were covered by ≥100 total reads and supported by ≥4 variant reads in the tumor and in the matched normal sample covered by ≥50 total reads and supported by ≤2 variant reads. ANNOVAR and TransVar were used for annotation with public variant databases [25, 26]. Variants were filtered if the baseline population frequency ≥ 5%.

### 2.6. Statistical Analysis

Nonparametric statistical tests were used throughout analysis due to a small sample size, and where *P* values were determined, the significance level was set at 0.05. Data for qualitative variables were reported as median and range. The association of clinical stage with clinical values, i.e., age and serum levels of protein biomarker, was analyzed as a continuous variable using the Wilcoxon rank sum test, while sex was analyzed as a categorial variable using the Fisher's exact test. The association between NSE levels and mutation counts was assessed using Spearman's correlation test. Statistical analysis was performed using R version 3.6.0.

## 3. Results

### 3.1. Baseline Characteristics

Table 1 shows the clinical characteristics of 32 Chinese SCLC patients. They tended to be older, at 65 years of age, and male. Under TNM classification, T2 and N0 were the most common categories (Table S1). We further classified patients into two broad clinical staging groups, stage I (*n* = 16) versus stages II + III (*n* = 16), to examine whether there were statistical differences between cancer stage and clinical values. Among the four investigated serum protein biomarkers, we found that serum levels of NSE of patients at stages II + III were significantly higher compared to stage I (*P* = 7 × 10^–4^; Table 1 and Figure S1).

### 3.2. Identification of SCLC-Related Oncogenic Genes

**Figure 1:**
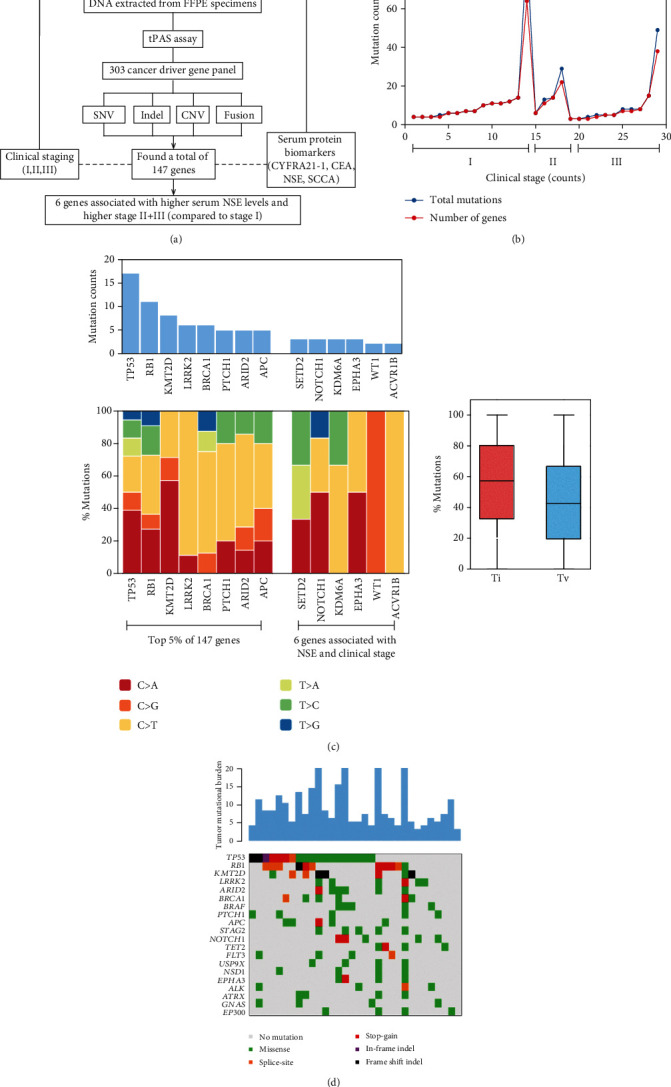
Study overview: (a) sequencing and analytical pipeline; (b) total mutations and number of genes per patient arranged according to clinical stages I, II, and III; (c) mutation counts of top 5% SCLC-related mutated genes observed in 90% of patients and 6 genes associated with NSE and clinical stage, where mutation to same gene is counted once (top) with their individual and combined summary of transition (Ti) and transversion (Tv) mutations (bottom); (d) mutational landscape of SCLC showing the number of coding somatic mutations per megabase of DNA (top) and a matrix of 20 frequently mutated genes colored by the type of variation (bottom).

An overview of this descriptive case study is shown in Figure 1(a). From the 32 SCLC patients, we detected a total of 147 oncogenes. Each patient harbored an average of 12.6 (range: 3, 90) pathogenic mutations. Total mutations and genes across patients arranged by clinical stage are shown in Figure 1(b). Table S2 shows the list of 147 SCLC-related genes ranked by total mutation counts. After we ranked the genes according to mutation number, we found *TP53*, *RB1*, and *KMT2D* were the most frequently mutated genes (representing top 2% of all genes, observed in 79% of patients). Including five more genes that shared same number of mutations, *LRRK2*, *BRCA1*, *PTCH1*, *ARID2*, and *APC*, increased the representation to top 5% of all genes, observed in 90% of patients. Together, these eight genes were the cut-off for the commonly mutated genes in this Chinese population of SCLC patients (Figure 1(c)).

### 3.3. Profiling of SNVs, Indels, and CNVs

The most common nucleotide mutations to the genes *TP53*, *RB1*, and *KMT2D* were C > A, C > T, and C > A transitions, respectively (Figure 1(c)). Total number of mutations per coding area of a tumor genome (tumor mutational burden (TMB)) ranged between 3 and 20 (Figure 1(d)). The mutational spectra of SCLC were dominated by missense, where indel and *frameshift* mutations occurred predominantly in *TP53*, *RB1*, and *KMT2D*. There were two mutation hotspots, c.832C > T (p.P278S) and c.1548G > A (p.W516X), observed in *TP53* and RB1, respectively (Table S2). No CNV mutations were found in any of the genes.

### 3.4. SCLC Gene Variant Association with Serum Biomarkers and Clinical Stage

**Figure 2:**
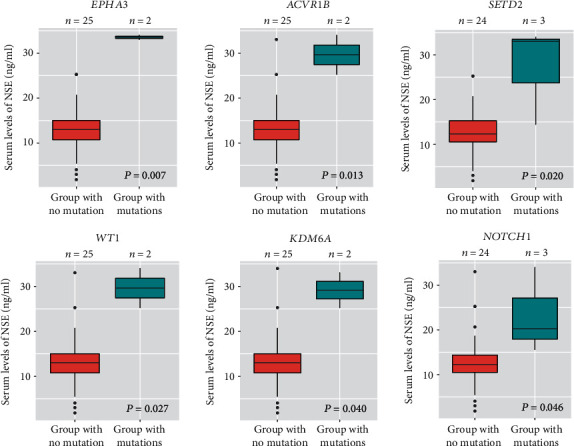
Significantly higher serum levels of NSE in SCLC patients with mutations to the following 6 genes (Wilcoxon rank sum test *P* = 0.007–0.046).

**Figure 3:**
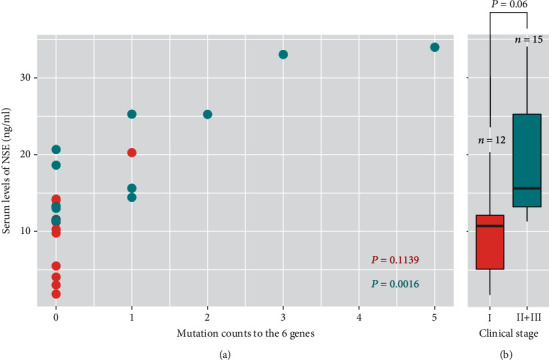
Increased mutations to 6 genes are associated with higher serum levels of NSE and clinical stage of SCLC. Scatterplot (a) shows patients with increased mutations to the following 6 genes (*WT1*, *NOTCH1*, *EPHA3*, *KDM6A*, *SETD2*, *ACVR1B*) have significantly higher NSE levels (Spearman's correlation test *P* = 0.0016) compared to those with ≤1 mutation to these 6 genes (*P* = 0.1139). Boxplot (b) shows patients with increased mutations to these 6 genes also have a significantly higher clinical stages II + III compared to stage I (Wilcoxon rank sum test *P* = 0.06).

Because NSE associated with clinical stage, we also searched among the 147 genes for those that associated with NSE levels. We found 6 genes (*WT1*, *NOTCH1*, *EPHA3*, *KDM6A*, *SETD2*, *ACVR1B*) associated with serum NSE levels (*P* = 0.007–0.046; Figure 2). Generally, the more mutations to these 6 genes, the higher were the NSE levels (*P* = 0.0016) compared with one or no mutation to these 6 genes that were not associated with NSE levels (*P* = 0.1139), and furthermore, these 6 genes were associated with clinical stages II + III compared to I (marginal *P* = 0.06, Figure 3).

## 4. Discussion

From 32 Chinese SCLC patients, we detected 147 out of 303 cancer driver oncogenes, including three previously implicated genes, *TP53*, *RB1*, and *KMT2D*, that appeared in 79% of our patients across all clinical stage. Together with *LRRK2*, *BRCA1*, *PTCH1*, *ARID2*, and *APC*, these eight genes represented top 5% of all genes that occurred in 90% of patients. In this study, we further found that serum protein marker NSE significantly associated with higher clinical stages II + III (*P* = 7 × 10^–4^). In particular, increased mutations to 6 genes (*WT1*, *NOTCH1*, *EPHA3*, *KDM6A*, *SETD2*, *ACVR1B*) significantly associated with higher NSE levels (*P* = 0.0016) and stages II + III (*P* = 0.06).

Loss of functions to *TP53* and *RB1* highly associate with the pathogenesis of SCLC [27] and are found in 82% and 62% of SCLC patients, respectively [28]. Previous study indicates that G:C to T:A transversions are the most frequently observed nucleotide substitutions to *TP53* in lung cancer [29], which is consistent with our finding. In contrast, *KMT2D* mutations are less related to *TP53* and *RB1*; it has instead been linked to a longer survival in patients with lung neuroendocrine tumors [30] despite that it has also been shown not to be involved in reducing survival of NSCLC patients [31]. In our study, the third most frequent gene mutations were in the *KMT2D* gene, whether the *KMT2D* gene plays a crucial role in survival of Chinese SCLC patient needs to be further investigated.

Among the five novel SCLC-related mutated genes found in this study, the *LRRK2* is a mutation gene in the Chinese population with familial Parkinson's disease [32]. *LRRK2* mutation carriers have an increased risk of cancer, especially for hormone-related cancer, and SCLC is associated with polypeptide hormone production [33]. The *BRCA1* is involved in tumor suppression and homologous recombination repair in response to DNA breaks and may be a modulator of mitotic spindle assembly [34, 35]. Whole exome sequencing of the lung cancer has revealed a germline *BRCA1* deficiency mutational signature [36]. We have identified mutations in *LRRK2* and *BRCA1* genes, and this represents the first report in the Chinese patients with SCLC. Finally, *ARID2* is a tumor suppressor gene, where inactivating mutations along the *ARID2* coding region is also detected in NSCLC [37]. *ARID2* knockout results in dysfunction of DNA repair process, leading to susceptibility to carcinogens in human hepatocellular carcinoma cells [38]. However, it is unclear how *ARID2* mutations are associated with SCLC progression. We identified a 46.9% variant allele fraction to c.3004A > G (p.T1002A) in *ARID2* of a SCLC patient. To our knowledge, our data is the first to report such high frequency SNV of *ARID2* gene in SCLC.

In this study, serum levels of NSE associated with higher clinical stage of SCLC. It has been reported that NSE (81.2%) has the highest sensitivity, followed by CEA (42.7%), CYFRA21-1 (32.3%), and SCCA (1%), and the average concentration of NSE was statistically higher in the patients with extensive disease (88.2%) compared to limited disease (73.3%) [39]. Recent studies, such as CancerSEEK, used 16 gene mutations combined with abnormal levels of 8 protein biomarkers for early diagnosis of lung cancer [40]. Similarly, we found 6 mutated genes that could associate with serum NSE to provide information about the severity of SCLC.

Some limitations of this study should be noted. First, we focused on SNV and indels as their function could be predicted by numerous methods and that heterogeneity has immediate clinical consequences for treatment selection [41]. Second, we could not evaluate recurrent noncoding, copy-number, or epigenetic mutations because the functional prediction methods for them were not yet available. Third, we did not assess micrometastases that are not visible clinically, so could not exclude those mutations in yet undiscovered driver genes of metastases. Finally, the top 5% mutated genes did not overlap with the 6 genes associated with NSE and stage, might be the small sample size, or that 6 genes represented a subpopulation of SCLC patients.

## 5. Conclusion

About 50% of oncogenic driver mutations are detected by our panel, suggesting it is moderately enriched for SCLC detection. Apart from capturing the most important SCLC-related mutant genes reported in other studies, we further found 5 genes and increased mutations to 6 genes associated with higher NSE and clinical stage. More investigations are required to evaluate the possibility of combining these mutant genes with NSE into a fast and simple companion diagnostic kit for clinicians to detect SCLC stage.

## Figures and Tables

**Table 1:** Clinical characteristics of SCLC patients.

Parameters	Clinical values^∗^	Stage I	Stages II + III	*P* value^∗∗^
Age (years old)	65 (49, 81)	67 (51, 81)	63 (49, 81)	0.2506
Sex (male/female)	24/8	12/4	12/4	0.9999
CYFRA21-1 (ng/ml)	4.94 (1.24, 19.53)	3.41 (1.24, 19.53)	3.07 (2.04, 4.43)	0.5033
CEA (ng/ml)	4.33 (0.83, 18.37)	2.97 (0.83, 16.59)	2.52 (1.37, 18.37)	0.9786
NSE (ng/ml)	14.37 (1.83, 34.00)	10.74 (1.83, 20.26)	15.60 (11.38, 34.00)	7.56 × 10^–4^
SCCA (ng/ml)	0.97 (0.20, 3.08)	0.88 (0.20, 1.83)	0.86 (0.21, 3.08)	0.9742

^∗^Reported are median and range, except for sex is total individuals. ^∗∗^*P* values for age, CYFRA21-1, CEA, NSE, and SCCA were generated by Wilcoxon rank sum test; *P* value for sex was generated by Fisher's exact test.

## Data Availability

The data are available upon the authors' reasonable request.

## Supplementary Materials

Supplementary Materials Figure S1: serum levels of NSE significantly associated with a higher clinical stages II + III of SCLC (Wilcoxon rank sum test *P* = 7 × 10^–4^). Table S1: clinical category IASLC T1–4 (any N) M0 or N0–N3 (any T) M0 SCLC. Table S2: list of 147 SCLC-related mutant genes ranked by total mutation counts.
