# Supplementary material for: Genetic Mutation Analysis in Small Cell Lung Cancer by a Novel NGS-Based Targeted Resequencing Gene Panel and Relation with Clinical Features
Source: Biomed Res Int. 2021 Apr 5;2021:3609028. doi: 10.1155/2021/3609028 (PMC8046547; doi:10.1155/2021/3609028)
Supplement: Supplementary Materials — Figure S1: serum levels of NSE significantly associated with a higher clinical stages II + III of SCLC (Wilcoxon rank sum test P = 7 × 10^–4^). Table S1: clinical category IASLC T1–4 (any N) M0 or N0–N3 (any T) M0 SCLC. Table S2: list of 147 SCLC-related mutant genes ranked by total mutation counts. [file 3609028.f1.pdf]

Figure S1. Serum levels of NSE significantly associated with a higher clinical stage II+III of SCLC (Wilcoxon rank sum test P=7×10^{-4})

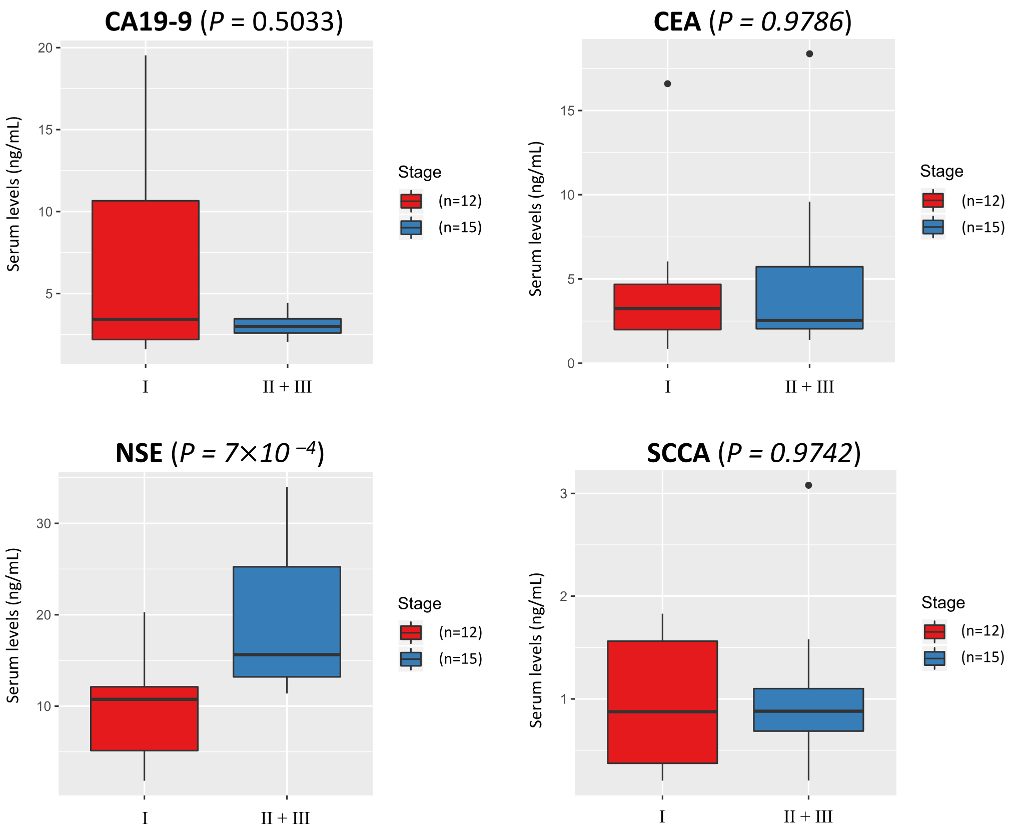

Table S1. Clinical Category IASLC T1–4 (Any N) M0 or N0–N3 (Any T) M0 SCLC.

Table S1. Data for Clinical Category IASLC T1–4 (Any N) M0 or N0–N3 (Any T) M0 SCLC			
**T Category**	**n**	**N Category**	**n**
T1	13	N0	16
T2	14	N1	6
T3	1	N2	10
T4	4	N3	0
IASLC, Internal Association for the Study of Lung Cancer. n, number			

Abbreviation: IASLC, Internal Association for the Study of Lung Cancer. n, number.

Table S2. List of 147 SCLC-related mutant genes ranked by total mutation counts

Gene	Chromosome	Transcript_ID	Exon
TP53	17	NM_000546	exon4
TP53	17	NM_000546	exon4
TP53	17	NM_000546	exon4
TP53	17	NM_000546	exon5
TP53	17	NM_000546	exon5
TP53	17	NM_000546	exon5
TP53	17	NM_000546	exon5
TP53	17	NM_000546	exon5
TP53	17	NM_000546	exon6
TP53	17	NM_000546	exon6
TP53	17	NM_000546	exon7
TP53	17	NM_000546	exon7
TP53	17	NM_000546	exon8
TP53	17	NM_000546	exon8
TP53	17	NM_000546	exon8
TP53	17	NM_000546	exon8
TP53	17	NM_000546	exon8
TP53	17	NM_000546	exon8
TP53	17	NM_000546	exon8
TP53	17	NM_000546	exon9
TP53	17	NM_001126113	exon11
RB1	13	NM_000321	exon2
RB1	13	NM_000321	exon2
RB1	13	NM_000321	exon11
RB1	13	NM_000321	exon11
RB1	13	NM_000321	exon16
RB1	13	NM_000321	exon17
RB1	13	NM_000321	exon17
RB1	13	NM_000321	exon17
RB1	13	NM_000321	exon20
RB1	13	NM_000321	exon20
RB1	13	NM_000321	exon24
RB1	13	NM_000321	exon24
KMT2D	12	NM_003482	exon4
KMT2D	12	NM_003482	exon5
KMT2D	12	NM_003482	exon9
KMT2D	12	NM_003482	exon29
KMT2D	12	NM_003482	exon31
KMT2D	12	NM_003482	exon44
KMT2D	12	NM_003482	exon44

KMT2D	12	NM_003482	exon45
KMT2D	12	NM_003482	exon52
KMT2D	12	NM_003482	exon53
LRRK2	12	NM_198578	exon9
LRRK2	12	NM_198578	exon15
LRRK2	12	NM_198578	exon22
LRRK2	12	NM_198578	exon22
LRRK2	12	NM_198578	exon29
LRRK2	12	NM_198578	exon30
LRRK2	12	NM_198578	exon32
LRRK2	12	NM_198578	exon49
LRRK2	12	NM_198578	exon51
BRCA1	17	NM_007300	exon3
BRCA1	17	NM_007294	exon10
BRCA1	17	NM_007294	exon10
BRCA1	17	NM_007294	exon10
BRCA1	17	NM_007294	exon10
BRCA1	17	NM_007294	exon10
BRCA1	17	NM_007294	exon10
BRCA1	17	NM_007294	exon19
PTCH1	9	NM_001083603	exon1
PTCH1	9	NM_000264	exon16
PTCH1	9	NM_000264	exon16
PTCH1	9	NM_000264	exon23
PTCH1	9	NM_000264	exon23
ARID2	12	NM_152641	exon11
ARID2	12	NM_152641	exon13
ARID2	12	NM_152641	exon15
ARID2	12	NM_152641	exon15
ARID2	12	NM_152641	exon15
ARID2	12	NM_152641	exon17
ARID2	12	NM_152641	exon21
APC	5	NM_000038	exon4
APC	5	NM_000038	exon7
APC	5	NM_000038	exon10
APC	5	NM_000038	exon15
APC	5	NM_000038	exon16
TET2	4	NM_017628	exon3
TET2	4	NM_017628	exon3
TET2	4	NM_001127208	exon3
TET2	4	NM_001127208	exon11

TAF1	X	NM_004606	exon6
TAF1	X	NM_004606	exon6
TAF1	X	NM_004606	exon9
TAF1	X	NM_004606	exon29
TAF1	X	NM_004606	exon37
STAG2	X	NM_001282418	exon4
STAG2	X	NM_001282418	exon4
STAG2	X	NM_001282418	exon6
STAG2	X	NM_001282418	exon9
STAG2	X	NM_001282418	exon10
STAG2	X	NM_001282418	exon13
STAG2	X	NM_001282418	exon19
NSD1	5	NM_022455	exon5
NSD1	5	NM_022455	exon5
NSD1	5	NM_022455	exon19
NSD1	5	NM_022455	exon23
KMT2C	7	NM_170606	exon2
KMT2C	7	NM_170606	exon5
KMT2C	7	NM_170606	exon36
KMT2C	7	NM_170606	exon38
KMT2C	7	NM_170606	exon43
KMT2C	7	NM_170606	exon48
GNAS	20	NM_001077490	exon1
GNAS	20	NM_016592	exon1
GNAS	20	NM_080425	exon1
GNAS	20	NM_001077490	exon1
FLT3	13	NM_004119	exon3
FLT3	13	NM_004119	exon7
FLT3	13	NM_004119	exon7
FLT3	13	NM_004119	exon13
EP300	22	NM_001429	exon13
EP300	22	NM_001429	exon28
EP300	22	NM_001429	exon31
EP300	22	NM_001429	exon31
BRAF	7	NM_004333	exon1
BRAF	7	NM_004333	exon1
BRAF	7	NM_004333	exon1
BRAF	7	NM_004333	exon1
BRAF	7	NM_004333	exon4
ATRX	X	NM_000489	exon9
ATRX	X	NM_000489	exon9

ATRX	X	NM_000489	exon9
ATRX	X	NM_000489	exon9
ATRX	X	NM_000489	exon9
ATRX	X	NM_000489	exon14
ATRX	X	NM_000489	exon16
ATRX	X	NM_000489	exon20
ATRX	X	NM_000489	exon35
ATM	11	NM_000051	exon13
ATM	11	NM_000051	exon20
ATM	11	NM_000051	exon44
ATM	11	NM_000051	exon50
ALK	2	NM_004304	exon6
ALK	2	NM_004304	exon7
ALK	2	NM_004304	exon28
ALK	2	NM_004304	exon29
XPO1	2	NM_003400	exon5
XPO1	2	NM_003400	exon18
XPO1	2	NM_003400	exon23
XPO1	2	NM_003400	exon23
USP9X	X	NM_001039590	exon8
USP9X	X	NM_001039590	exon12
USP9X	X	NM_001039590	exon14
USP9X	X	NM_001039590	exon19
USP9X	X	NM_001039590	exon24
USP9X	X	NM_001039590	exon35
SUFU	10	NM_016169	exon4
SUFU	10	NM_016169	exon7
SUFU	10	NM_016169	exon8
SUFU	10	NM_016169	exon12
SMARCA4	19	NM_001128849	exon12
SMARCA4	19	NM_001128849	exon17
SMARCA4	19	NM_001128849	exon26
SETD2	3	NM_014159	exon11
SETD2	3	NM_014159	exon11
SETD2	3	NM_014159	exon12
ROS1	6	NM_002944	exon11
ROS1	6	NM_002944	exon17
ROS1	6	NM_002944	exon21
ROS1	6	NM_002944	exon26
ROS1	6	NM_002944	exon26
PTEN	10	NM_000314	exon1

PTEN	10	NM_000314	exon6
PTEN	10	NM_000314	exon7
PIK3CA	3	NM_006218	exon10
PIK3CA	3	NM_006218	exon10
PIK3CA	3	NM_006218	exon12
PIK3CA	3	NM_006218	exon21
PBRM1	3	NM_018313	exon11
PBRM1	3	NM_018313	exon15
PBRM1	3	NM_018313	exon18
PBRM1	3	NM_018313	exon27
NOTCH1	9	NM_017617	exon7
NOTCH1	9	NM_017617	exon11
NOTCH1	9	NM_017617	exon12
NOTCH1	9	NM_017617	exon13
NOTCH1	9	NM_017617	exon18
NOTCH1	9	NM_017617	exon18
NFE2L2	2	NM_006164	exon5
NFE2L2	2	NM_006164	exon5
NFE2L2	2	NM_006164	exon5
MSH2	2	NM_000251	exon6
MSH2	2	NM_000251	exon10
MSH2	2	NM_000251	exon14
KMT2B	19	NM_014727	exon3
KMT2B	19	NM_014727	exon3
KMT2B	19	NM_014727	exon28
KDM6A	X	NM_001291415	exon16
KDM6A	X	NM_001291415	exon27
KDM6A	X	NM_001291416	exon28
IDH1	2	NM_005896	exon3
IDH1	2	NM_001282386	exon6
IDH1	2	NM_001282386	exon9
FBXW7	4	NM_033632	exon3
FBXW7	4	NM_033632	exon5
FBXW7	4	NM_033632	exon9
EPHA3	3	NM_005233	exon6
EPHA3	3	NM_005233	exon7
EPHA3	3	NM_005233	exon9
EPHA3	3	NM_005233	exon12
BRCA2	13	NM_000059	exon6
BRCA2	13	NM_000059	exon7
BRCA2	13	NM_000059	exon11

BRCA2	13	NM_000059	exon15
BRCA2	13	NM_000059	exon18
BRCA2	13	NM_000059	exon27
ATR	3	NM_001184	exon4
ATR	3	NM_001184	exon9
ATR	3	NM_001184	exon37
ATR	3	NM_001184	exon37
ATR	3	NM_001184	exon37
ATR	3	NM_001184	exon37
ATR	3	NM_001184	exon45
ATR	3	NM_001184	exon46
ABL1	9	NM_007313	exon7
ABL1	9	NM_007313	exon10
ABL1	9	NM_007313	exon11
WT1	11	NM_024426	exon4
WT1	11	NM_024426	exon7
TSHR	14	NM_000369	exon2
TSHR	14	NM_000369	exon10
TET1	10	NM_030625	exon2
TET1	10	NM_030625	exon12
TEK	9	NM_000459	exon6
TEK	9	NM_000459	exon21
RUNX1	21	NM_001754	exon3
RUNX1	21	NM_001754	exon4
RUNX1	21	NM_001754	exon4
RAD50	5	NM_005732	exon8
RAD50	5	NM_005732	exon12
RAD50	5	NM_005732	exon17
RAD50	5	NM_005732	exon18
PTPRD	9	NM_002839	exon39
PTPRD	9	NM_002839	exon40
POLQ	3	NM_199420	exon15
POLQ	3	NM_199420	exon16
PDGFRB	5	NM_002609	exon5
PDGFRB	5	NM_002609	exon13
PDGFRB	5	NM_002609	exon22
PALB2	16	NM_024675	exon4
PALB2	16	NM_024675	exon4
PALB2	16	NM_024675	exon12
NTRK3	15	NM_001012338	exon7
NTRK3	15	NM_001012338	exon15

NOTCH4	6	NM_004557	exon3
NOTCH4	6	NM_004557	exon24
NFE2L3	7	NM_004289	exon4
NFE2L3	7	NM_004289	exon4
NCOR1	17	NM_006311	exon16
NCOR1	17	NM_006311	exon27
NAV3	12	NM_014903	exon2
NAV3	12	NM_014903	exon15
NAV3	12	NM_014903	exon15
NAV3	12	NM_014903	exon24
MEN1	11	NM_130799	exon2
MEN1	11	NM_130799	exon9
LYN	8	NM_002350	exon2
LYN	8	NM_002350	exon4
KDR	4	NM_002253	exon4
KDR	4	NM_002253	exon13
JAK1	1	NM_002227	exon10
JAK1	1	NM_002227	exon25
ITK	5	NM_005546	exon9
ITK	5	NM_005546	exon11
HRAS	11	NM_005343	exon2
HRAS	11	NM_005343	exon2
GNAQ	9	NM_002072	exon3
GNAQ	9	NM_002072	exon5
FOXP1	3	NM_032682	exon12
FOXP1	3	NM_032682	exon13
FOXP1	3	NM_032682	exon18
FOXP1	3	NM_032682	exon21
EZH2	7	NM_004456	exon6
EZH2	7	NM_004456	exon9
ETV1	7	NM_004956	exon4
ETV1	7	NM_004956	exon13
EPHA2	1	NM_004431	exon4
EPHA2	1	NM_004431	exon6
DNMT3A	2	NM_022552	exon3
DNMT3A	2	NM_022552	exon15
CTNNB1	3	NM_001904	exon6
CTNNB1	3	NM_001904	exon11
CRIPAK	4	NM_175918	exon1
CRIPAK	4	NM_175918	exon1
CREBBP	16	NM_004380	exon26

CREBBP	16	NM_004380	exon26
CDKN2A	9	NM_000077	exon2
CDKN2A	9	NM_000077	exon2
CDK12	17	NM_015083	exon11
CDK12	17	NM_015083	exon14
BRIP1	17	NM_032043	exon7
BRIP1	17	NM_032043	exon15
BRIP1	17	NM_032043	exon20
BLM	15	NM_000057	exon3
BLM	15	NM_000057	exon7
BLM	15	NM_000057	exon20
ARID1A	1	NM_006015	exon2
ARID1A	1	NM_006015	exon2
AR	X	NM_000044	exon1
AR	X	NM_000044	exon1
ACVR1B	12	NM_020328	exon6
ACVR1B	12	NM_020328	exon9
TYRO3	15	NM_006293	exon3
TSC2	16	NM_001077183	exon5
TLR4	9	NM_138554	exon3
TBX3	12	NM_016569	exon7
SMO	7	NM_005631	exon2
SMO	7	NM_005631	exon2
SMC3	10	NM_005445	exon10
SMC3	10	NM_005445	exon13
SMC3	10	NM_005445	exon24
SMC1A	X	NM_006306	exon14
SMAD4	18	NM_005359	exon6
SIN3A	15	NM_001145357	exon6
SIK1	21	NM_173354	exon8
SF3B1	2	NM_012433	exon5
SDHC	1	NM_003001	exon6
REL	2	NM_002908	exon11
RECQL4	8	NM_004260	exon12
RECQL4	8	NM_004260	exon20
RARA	17	NM_000964	exon7
RAD21	8	NM_006265	exon10
PTPN11	12	NM_002834	exon6
PIK3R1	5	NM_181523	exon4
PARP3	3	NM_001003931	exon4
NF2	22	NM_181829	exon8

NF1	17	NM_001042492	exon6
NF1	17	NM_001042492	exon38
MTOR	1	NM_004958	exon41
MSH6	2	NM_000179	exon4
MRE11A	11	NM_005591	exon5
MRE11A	11	NM_005591	exon10
MLH1	3	NM_000249	exon18
MCL1	1	NM_182763	exon2
MAPK8IP1	11	NM_005456	exon9
MAP2K4	17	NM_001281435	exon7
LIMK1	7	NM_002314	exon14
LCK	1	NM_005356	exon10
KRAS	12	NM_004985	exon2
KITLG	12	NM_000899	exon7
IRS2	13	NM_003749	exon1
IL7R	5	NM_002185	exon8
IKZF1	7	NM_006060	exon7
HIST1H1C	6	NM_005319	exon1
HGF	7	NM_000601	exon17
HDAC6	X	NM_006044	exon2
HDAC3	5	NM_003883	exon4
HDAC2	6	NM_001527	exon11
GATA1	X	NM_002049	exon3
GATA1	X	NM_002049	exon3
FLT1	13	NM_002019	exon12
FLT1	13	NM_002019	exon15
FLCN	17	NM_144997	exon10
FGFR4	5	NM_213647	exon18
FGFR2	10	NM_000141	exon14
FANCC	9	NM_000136	exon13
ERG	21	NM_182918	exon1
ERG	21	NM_001291391	exon8
ERG	21	NM_001243432	exon11
ERCC5	13	NM_001204425	exon15
ERCC4	16	NM_005236	exon4
ERBB3	12	NM_001982	exon28
EPPK1	8	NM_031308	exon2
EPHB6	7	NM_004445	exon4
EPHB1	3	NM_004441	exon3
EPHA1	7	NM_005232	exon18
DICER1	14	NM_177438	exon10

CSF1R	5	NM_005211	exon16
CRKL	22	NM_005207	exon2
CDKN1A	6	NM_078467	exon3
CDK8	13	NM_001260	exon12
CDK6	7	NM_001259	exon2
CDH1	16	NM_004360	exon5
CBFB	16	NM_022845	exon1
CBFB	16	NM_022845	exon1
BTK	X	NM_000061	exon3
BMPR1A	10	NM_004329	exon7
BAP1	3	NM_004656	exon14
AURKA	20	NM_198433	exon7
ASXL1	20	NM_015338	exon12
ARHGAP35	19	NM_004491	exon1
AMER1	X	NM_152424	exon2
AKT1	14	NM_005163	exon14

DNA_Change	Amino_Acid_Change	Region
c.267delC	p.S90Pfs	exonic
c.329G>T	p.R110L	exonic
c.193A>T	p.R65X	exonic
c.463_468delACCCGC	p.T155_R156delTR	exonic
c.536A>G	p.H179R	exonic
c.503A>C	p.H168P	exonic
c.473G>T	p.R158L	exonic
c.456_465delGCCCGGCACC	p.P153Afs	exonic
c.596G>T	p.G199V	exonic
c.587G>C	p.R196P	exonic
c.743G>T	p.R248L	exonic
c.722C>T	p.S241F	exonic
c.832C>T	p.P278S	exonic
c.826G>C	p.A276P	exonic
c.892G>T	p.E298X	exonic
c.832C>T	p.P278S	exonic
c.854A>T	p.E285V	exonic
c.839G>T	p.R280I	exonic
c.818G>T	p.R273L	exonic
c.949C>T	p.Q317X	exonic
c.1054-2A>G	.	splicing
c.264+1G>T	.	splicing
c.163C>T	p.P55S	exonic
c.1059delA	p.Q354Rfs	exonic
c.1073G>A	p.R358Q	exonic
c.1498+1G>T	.	splicing
c.1548G>A	p.W516X	exonic
c.1499-2A>G	.	splicing
c.1548G>A	p.W516X	exonic
c.2076T>G	p.Y692X	exonic
c.1961-2A>G	.	splicing
c.2501C>G	p.S834X	exonic
c.2520+1G>T	.	splicing
c.401-1G>A	.	splicing
c.562G>T	p.G188X	exonic
c.1133delG	p.G378Afs	exonic
c.6110-1G>T	.	splicing
c.6411_6420delCTCTGCGGAC	p.S2138Gfs	exonic
c.14145G>T	p.L4715F	exonic
c.14120C>T	p.P4707L	exonic

c.14260G>T	p.D4754Y	exonic
c.16384G>C	p.D5462H	exonic
c.16509delC	p.G5505Efs	exonic
c.1015G>A	p.D339N	exonic
c.1742C>T	p.S581F	exonic
c.2878+1G>A	.	splicing
c.2861C>T	p.S954F	exonic
c.3960-1G>A	.	splicing
c.4273C>T	p.Q1425X	exonic
c.4639G>T	p.V1547L	exonic
c.7186G>A	p.V2396I	exonic
c.7522G>A	p.E2508K	exonic
c.134+2T>G	.	splicing
c.2286A>T	p.R762S	exonic
c.3448C>T	p.P1150S	exonic
c.1450G>A	p.G484R	exonic
c.2683C>T	p.Q895X	exonic
c.2258G>A	p.S753N	exonic
c.962G>A	p.W321X	exonic
c.5215G>C	p.D1739H	exonic
c.32T>C	p.I11T	exonic
c.2678G>A	p.R893H	exonic
c.2692G>A	p.D898N	exonic
c.3913G>T	p.D1305Y	exonic
c.4127C>T	p.S1376F	exonic
c.1445G>C	p.R482T	exonic
c.1640C>T	p.S547F	exonic
c.3004A>G	p.T1002A	exonic
c.4304C>T	p.S1435L	exonic
c.4336G>T	p.D1446Y	exonic
c.5011G>A	p.G1671S	exonic
c.5467C>T	p.Q1823X	exonic
c.307G>A	p.V103I	exonic
c.706C>T	p.Q236X	exonic
c.1276G>T	p.A426S	exonic
c.1910G>C	p.G637A	exonic
c.4451A>G	p.D1484G	exonic
c.3478G>A	p.G1160R	exonic
c.3451G>T	p.E1151X	exonic
c.2398C>T	p.H800Y	exonic
c.4997C>T	p.P1666L	exonic

c.885G>T	p.Q295H	exonic
c.885G>T	p.Q295H	exonic
c.1582G>A	p.E528K	exonic
c.4457C>A	p.S1486X	exonic
c.5303G>A	p.G1768E	exonic
c.G91A	p.E31K	exonic
c.C68T	p.S23F	exonic
c.C349T	p.L117F	exonic
c.C811T	p.R271W	exonic
c.A890G	p.Y297C	exonic
c.A1118T	p.D373V	exonic
c.T1783C	p.F595L	exonic
c.2592G>T	p.L864F	exonic
c.2677G>A	p.A893T	exonic
c.5942G>A	p.C1981Y	exonic
c.6991G>A	p.D2331N	exonic
c.175G>A	p.G59R	exonic
c.695G>A	p.G232E	exonic
c.6373C>T	p.P2125S	exonic
c.8167G>T	p.D2723Y	exonic
c.11414C>T	p.T3805I	exonic
c.12244G>C	p.V4082L	exonic
c.441G>C	p.R147S	exonic
c.467G>A	p.G156D	exonic
c.293C>T	p.P98L	exonic
c.98C>G	p.A33G	exonic
c.368+1G>A	.	splicing
c.834C>G	p.N278K	exonic
c.834C>G	p.N278K	exonic
c.1598G>T	p.G533V	exonic
c.2348C>T	p.A783V	exonic
c.4567G>A	p.E1523K	exonic
c.6354G>T	p.M2118I	exonic
c.5510G>A	p.R1837Q	exonic
c.68T>G	p.M23R	exonic
c.68T>G	p.M23R	exonic
c.68T>G	p.M23R	exonic
c.68T>G	p.M23R	exonic
c.587C>T	p.A196V	exonic
c.2120C>T	p.A707V	exonic
c.1042G>A	p.V348M	exonic

c.803G>A	p.C268Y	exonic
c.3371C>T	p.S1124F	exonic
c.2477A>G	p.K826R	exonic
c.4307G>A	p.S1436N	exonic
c.4636A>G	p.K1546E	exonic
c.5219C>T	p.S1740L	exonic
c.7421G>A	p.R2474H	exonic
c.2044G>A	p.V682I	exonic
c.2933C>T	p.S978F	exonic
c.6397C>G	p.Q2133E	exonic
c.7432G>A	p.E2478K	exonic
c.1283-1G>A	.	splicing
c.1516C>A	p.L506I	exonic
c.4086G>A	p.M1362I	exonic
c.4573A>G	p.K1525E	exonic
c.338C>T	p.T113M	exonic
c.2059C>A	p.Q687K	exonic
c.2960C>G	p.P987R	exonic
c.2855delT	p.L952Wfs	exonic
c.859G>A	p.E287K	exonic
c.1594C>A	p.H532N	exonic
c.1852G>A	p.E618K	exonic
c.2728G>A	p.D910N	exonic
c.3635G>A	p.C1212Y	exonic
c.5876T>C	p.I1959T	exonic
c.502G>C	p.D168H	exonic
c.838C>T	p.R280W	exonic
c.964C>T	p.P322S	exonic
c.1445C>T	p.P482L	exonic
c.1847C>T	p.P616L	exonic
c.2441C>T	p.T814M	exonic
c.3574C>T	p.R1192C	exonic
c.5285A>T	p.H1762L	exonic
c.5365G>T	p.G1789C	exonic
c.5627T>C	p.L1876P	exonic
c.1216C>T	p.P406S	exonic
c.2515G>A	p.D839N	exonic
c.3248A>G	p.E1083G	exonic
c.4229C>T	p.A1410V	exonic
c.4238G>T	p.G1413V	exonic
c.79T>G	p.Y27D	exonic

c.571delG	p.V191Wfs	exonic
c.671T>G	p.I224R	exonic
c.1633G>A	p.E545K	exonic
c.1633G>A	p.E545K	exonic
c.1846G>T	p.V616F	exonic
c.3129G>T	p.M1043I	exonic
c.1043C>T	p.A348V	exonic
c.1741G>A	p.A581T	exonic
c.2608C>T	p.Q870X	exonic
c.4286C>G	p.P1429R	exonic
c.1132A>C	p.S378R	exonic
c.1862G>A	p.R621H	exonic
c.2014+1G>T	.	splicing
c.2146G>T	p.E716X	exonic
c.2864G>A	p.R955H	exonic
c.2830G>T	p.E944X	exonic
c.1346G>A	p.R449H	exonic
c.1346G>A	p.R449H	exonic
c.1346G>A	p.R449H	exonic
c.1024G>A	p.V342I	exonic
c.1600C>T	p.R534C	exonic
c.2408C>T	p.T803I	exonic
c.2398A>G	p.K800E	exonic
c.896G>A	p.G299D	exonic
c.6197A>C	p.D2066A	exonic
c.C1582T	p.H528Y	exonic
c.G3980A	p.W1327X	exonic
c.4197+2T>C	.	splicing
c.59G>A	p.R20Q	exonic
c.A623G	p.Y208C	exonic
c.C992T	p.A331V	exonic
c.527C>T	p.S176F	exonic
c.845C>T	p.S282L	exonic
c.1274G>A	p.W425X	exonic
c.1313C>A	p.S438X	exonic
c.1494C>A	p.S498R	exonic
c.1727C>T	p.A576V	exonic
c.2075G>A	p.S692N	exonic
c.500C>T	p.T167I	exonic
c.587G>A	p.S196N	exonic
c.3029G>C	p.R1010T	exonic

c.7522G>A	p.G2508S	exonic
c.7985C>T	p.T2662M	exonic
c.9737C>T	p.A3246V	exonic
c.1166C>T	p.A389V	exonic
c.1931C>T	p.P644L	exonic
c.6293G>A	p.G2098D	exonic
c.6266G>A	p.R2089Q	exonic
c.6292G>A	p.G2098S	exonic
c.6266G>A	p.R2089Q	exonic
c.7604C>T	p.A2535V	exonic
c.7739C>T	p.T2580I	exonic
c.1238C>T	p.T413I	exonic
c.1670C>T	p.A557V	exonic
c.2945C>T	p.T982I	exonic
c.942delC	p.L315Xfs	exonic
c.1138C>G	p.R380G	exonic
c.203C>T	p.P68L	exonic
c.982G>A	p.E328K	exonic
c.1528G>A	p.A510T	exonic
c.6085G>A	p.A2029T	exonic
c.776C>A	p.T259K	exonic
c.3136G>A	p.E1046K	exonic
c.88G>A	p.D30N	exonic
c.280A>T	p.S94C	exonic
c.253C>A	p.H85N	exonic
c.1208G>A	p.R403K	exonic
c.1855G>A	p.E619K	exonic
c.2758G>A	p.E920K	exonic
c.2884G>A	p.E962K	exonic
c.4619C>A	p.T1540N	exonic
c.4781A>G	p.Y1594C	exonic
c.2300A>G	p.N767S	exonic
c.4478G>C	p.S1493T	exonic
c.685G>A	p.E229K	exonic
c.1876C>T	p.Q626X	exonic
c.3044A>G	p.N1015S	exonic
c.1336G>A	p.D446N	exonic
c.212-1G>A	.	splicing
c.3241G>A	p.E1081K	exonic
c.493A>G	p.S165G	exonic
c.1699A>C	p.M567L	exonic

c.251T>C	p.L84P	exonic
c.4385T>C	p.L1462P	exonic
c.1121A>G	p.N374S	exonic
c.1806A>G	p.I602M	exonic
c.1670C>T	p.T557I	exonic
c.3670A>G	p.N1224D	exonic
c.337C>G	p.L113V	exonic
c.3073G>T	p.G1025X	exonic
c.3074G>T	p.G1025V	exonic
c.4933C>T	p.Q1645X	exonic
c.409C>G	p.R137G	exonic
c.1301T>G	p.V434G	exonic
c.52G>A	p.D18N	exonic
c.270G>A	p.M90I	exonic
c.446C>T	p.P149L	exonic
c.1819G>T	p.D607Y	exonic
c.1361G>A	p.R454Q	exonic
c.3444_3446delATT	p.F1149delF	exonic
c.836C>T	p.T279I	exonic
c.1003C>T	p.R335W	exonic
c.35G>A	p.G12D	exonic
c.38G>A	p.G13D	exonic
c.416C>T	p.P139L	exonic
c.671C>T	p.T224I	exonic
c.889C>T	p.P297S	exonic
c.1013C>T	p.T338I	exonic
c.1534G>A	p.A512T	exonic
c.1996G>T	p.D666Y	exonic
c.587G>C	p.R196T	exonic
c.913C>T	p.H305Y	exonic
c.113A>G	p.D38G	exonic
c.1148C>T	p.A383V	exonic
c.904G>T	p.E302X	exonic
c.1339C>T	p.R447C	exonic
c.112C>T	p.R38C	exonic
c.1690G>T	p.D564Y	exonic
c.884C>T	p.A295V	exonic
c.1690G>A	p.V564I	exonic
c.145C>T	p.P49S	exonic
c.1079A>G	p.E360G	exonic
c.4337G>T	p.R1446L	exonic

c.4318_4320delTTC	p.F1440delF	exonic
c.166A>G	p.S56G	exonic
c.172C>T	p.R58X	exonic
c.3091C>T	p.P1031S	exonic
c.4118A>T	p.H1373L	exonic
c.918+1G>A	.	splicing
c.2233G>A	p.A745T	exonic
c.3475G>A	p.A1159T	exonic
c.145G>A	p.V49I	exonic
c.1439C>T	p.S480F	exonic
c.3794G>A	p.G1265D	exonic
c.1244A>G	p.H415R	exonic
c.1244A>G	p.H415R	exonic
c.122A>C	p.H41P	exonic
c.392G>C	p.C131S	exonic
c.1094G>T	p.G365V	exonic
c.1454G>T	p.R485L	exonic
c.329C>T	p.S110F	exonic
c.406C>T	p.L136F	exonic
c.2446C>A	p.L816M	exonic
c.1420C>T	p.P474S	exonic
c.536C>T	p.T179M	exonic
c.536C>T	p.T179M	exonic
c.746G>A	p.S249N	exonic
c.1243G>A	p.A415T	exonic
c.2831G>T	p.R944L	exonic
c.2248G>A	p.D750N	exonic
c.668-1G>T	.	splicing
c.996G>T	p.L332F	exonic
c.779T>G	p.V260G	exonic
c.450G>C	p.R150S	exonic
c.432G>T	p.K144N	exonic
c.1441G>A	p.D481N	exonic
c.1915G>A	p.G639S	exonic
c.3329G>A	p.G1110E	exonic
c.898G>T	p.A300S	exonic
c.1201C>A	p.L401I	exonic
c.710C>T	p.A237V	exonic
c.490C>T	p.L164F	exonic
c.457C>T	p.H153Y	exonic
c.688-1G>A	.	splicing

c.587-2A>T	.	splicing
c.5490_5494delCTGGG	p.W1831Tfs	exonic
c.5715A>T	p.R1905S	exonic
c.2039C>T	p.A680V	exonic
c.346C>T	p.L116F	exonic
c.1096C>T	p.R366X	exonic
c.2041G>A	p.A681T	exonic
c.736C>T	p.Q246X	exonic
c.1864G>A	p.V622I	exonic
c.718+1G>A	.	splicing
c.1623+1G>A	.	splicing
c.971T>G	p.L324R	exonic
c.37G>T	p.G13C	exonic
c.676C>T	p.L226F	exonic
c.3552A>C	p.K1184N	exonic
c.1313T>C	p.L438P	exonic
c.C782T	p.S261F	exonic
c.566C>T	p.A189V	exonic
c.1911G>A	p.M637I	exonic
c.92C>A	p.S31X	exonic
c.328G>T	p.A110S	exonic
c.1147C>T	p.Q383X	exonic
c.283G>A	p.G95S	exonic
c.234C>A	p.Y78X	exonic
c.1589C>T	p.S530F	exonic
c.2173G>A	p.D725N	exonic
c.1133G>A	p.S378N	exonic
c.2356C>T	p.P786S	exonic
c.1977G>C	p.K659N	exonic
c.1297C>T	p.R433C	exonic
c.13A>G	p.I5V	exonic
c.G909T	p.L303F	exonic
c.940G>A	p.G314S	exonic
c.A2238G	p.I746M	exonic
c.787G>A	p.D263N	exonic
c.3634C>T	p.P1212S	exonic
c.2290A>C	p.T764P	exonic
c.331G>A	p.V111M	exonic
c.177G>C	p.Q59H	exonic
c.2857C>G	p.L953V	exonic
c.1586C>T	p.P529L	exonic

c.2138G>A	p.S713N	exonic
c.685C>T	p.P229S	exonic
c.184G>T	p.D62Y	exonic
c.1219G>T	p.V407F	exonic
c.2T>G	.	exonic
c.638G>A	p.W213X	exonic
c.20A>C	p.D7A	exonic
c.50T>G	p.F17C	exonic
c.206C>T	p.P69L	exonic
c.455G>A	p.R152Q	exonic
c.1750C>T	p.P584S	exonic
c.503A>G	p.Q168R	exonic
c.1910C>T	p.A637V	exonic
c.2725G>T	p.E909X	exonic
c.1634T>C	p.F545S	exonic
c.1436C>T	p.T479M	exonic

Mutation_Type	ReadDepth_Total	ReadDepth_Variant
frameshift deletion	160	119
nonsynonymous SNV	182	110
stopgain	157	40
nonframeshift deletion	1537	1359
nonsynonymous SNV	512	318
nonsynonymous SNV	1987	535
nonsynonymous SNV	1007	358
frameshift deletion	1307	1024
nonsynonymous SNV	838	376
nonsynonymous SNV	1247	345
nonsynonymous SNV	1474	527
nonsynonymous SNV	1479	255
nonsynonymous SNV	561	351
nonsynonymous SNV	849	419
stopgain	586	473
nonsynonymous SNV	421	109
nonsynonymous SNV	425	294
nonsynonymous SNV	1239	500
nonsynonymous SNV	430	354
stopgain	1137	738
.	506	340
.	310	192
nonsynonymous SNV	152	9
frameshift deletion	844	163
nonsynonymous SNV	152	10
.	1296	650
stopgain	1210	83
.	173	130
stopgain	213	47
stopgain	402	151
.	210	125
stopgain	303	212
.	237	135
.	612	198
stopgain	1408	788
frameshift deletion	1032	269
.	3018	1280
frameshift deletion	183	57
nonsynonymous SNV	784	381
nonsynonymous SNV	228	12

nonsynonymous SNV	155	41
nonsynonymous SNV	2187	786
frameshift deletion	1207	600
nonsynonymous SNV	266	14
nonsynonymous SNV	253	13
.	154	8
nonsynonymous SNV	207	13
.	179	11
stopgain	183	18
nonsynonymous SNV	790	212
nonsynonymous SNV	2136	118
nonsynonymous SNV	260	16
.	705	310
nonsynonymous SNV	2016	956
nonsynonymous SNV	2002	523
nonsynonymous SNV	179	9
stopgain	165	9
nonsynonymous SNV	163	9
stopgain	205	12
nonsynonymous SNV	2345	718
nonsynonymous SNV	1138	152
nonsynonymous SNV	1286	617
nonsynonymous SNV	716	137
nonsynonymous SNV	625	143
nonsynonymous SNV	151	10
nonsynonymous SNV	988	89
nonsynonymous SNV	264	16
nonsynonymous SNV	3416	1602
nonsynonymous SNV	196	10
nonsynonymous SNV	167	14
nonsynonymous SNV	218	25
stopgain	333	22
nonsynonymous SNV	216	14
stopgain	188	13
nonsynonymous SNV	269	48
nonsynonymous SNV	1092	491
nonsynonymous SNV	2293	233
nonsynonymous SNV	636	126
stopgain	1388	675
nonsynonymous SNV	269	14
nonsynonymous SNV	207	14

nonsynonymous SNV	997	865
nonsynonymous SNV	185	34
nonsynonymous SNV	407	21
stopgain	968	403
nonsynonymous SNV	180	10
nonsynonymous SNV	264	15
nonsynonymous SNV	198	14
nonsynonymous SNV	237	18
nonsynonymous SNV	161	9
nonsynonymous SNV	281	18
nonsynonymous SNV	273	83
nonsynonymous SNV	710	200
nonsynonymous SNV	1264	1043
nonsynonymous SNV	226	12
nonsynonymous SNV	308	18
nonsynonymous SNV	404	200
nonsynonymous SNV	266	24
nonsynonymous SNV	158	10
nonsynonymous SNV	161	12
nonsynonymous SNV	544	348
nonsynonymous SNV	353	26
nonsynonymous SNV	2863	1280
nonsynonymous SNV	1109	414
nonsynonymous SNV	1460	335
nonsynonymous SNV	2091	799
nonsynonymous SNV	1382	557
.	2006	270
nonsynonymous SNV	2249	872
nonsynonymous SNV	340	26
nonsynonymous SNV	381	148
nonsynonymous SNV	155	8
nonsynonymous SNV	302	20
nonsynonymous SNV	925	221
nonsynonymous SNV	324	159
nonsynonymous SNV	555	76
nonsynonymous SNV	296	35
nonsynonymous SNV	354	44
nonsynonymous SNV	349	49
nonsynonymous SNV	189	12
nonsynonymous SNV	198	11
nonsynonymous SNV	155	11

nonsynonymous SNV	392	26
nonsynonymous SNV	230	33
nonsynonymous SNV	216	12
nonsynonymous SNV	444	304
nonsynonymous SNV	183	10
nonsynonymous SNV	424	33
nonsynonymous SNV	160	10
nonsynonymous SNV	315	18
nonsynonymous SNV	236	15
nonsynonymous SNV	204	14
nonsynonymous SNV	2603	2233
.	175	10
nonsynonymous SNV	1995	787
nonsynonymous SNV	574	242
nonsynonymous SNV	743	342
nonsynonymous SNV	216	14
nonsynonymous SNV	262	16
nonsynonymous SNV	1898	387
frameshift deletion	498	83
nonsynonymous SNV	256	29
nonsynonymous SNV	489	378
nonsynonymous SNV	151	8
nonsynonymous SNV	292	21
nonsynonymous SNV	354	22
nonsynonymous SNV	1327	100
nonsynonymous SNV	1576	873
nonsynonymous SNV	381	57
nonsynonymous SNV	800	116
nonsynonymous SNV	2148	349
nonsynonymous SNV	229	116
nonsynonymous SNV	419	22
nonsynonymous SNV	2709	328
nonsynonymous SNV	1129	541
nonsynonymous SNV	367	143
nonsynonymous SNV	157	8
nonsynonymous SNV	188	10
nonsynonymous SNV	224	12
nonsynonymous SNV	565	49
nonsynonymous SNV	276	14
nonsynonymous SNV	206	18
nonsynonymous SNV	961	844

frameshift deletion	514	178
nonsynonymous SNV	448	95
nonsynonymous SNV	154	38
nonsynonymous SNV	479	58
nonsynonymous SNV	976	150
nonsynonymous SNV	1735	246
nonsynonymous SNV	213	11
nonsynonymous SNV	160	19
stopgain	225	14
nonsynonymous SNV	618	149
nonsynonymous SNV	1745	881
nonsynonymous SNV	1717	711
.	1780	335
stopgain	594	363
nonsynonymous SNV	1817	841
stopgain	2168	550
nonsynonymous SNV	2289	1174
nonsynonymous SNV	1357	72
nonsynonymous SNV	2146	116
nonsynonymous SNV	318	18
nonsynonymous SNV	240	35
nonsynonymous SNV	498	37
nonsynonymous SNV	2110	1105
nonsynonymous SNV	1024	530
nonsynonymous SNV	400	43
nonsynonymous SNV	168	12
stopgain	343	18
.	226	84
nonsynonymous SNV	162	100
nonsynonymous SNV	1070	516
nonsynonymous SNV	243	15
nonsynonymous SNV	170	10
nonsynonymous SNV	204	29
stopgain	157	19
stopgain	381	25
nonsynonymous SNV	1894	505
nonsynonymous SNV	354	18
nonsynonymous SNV	202	18
nonsynonymous SNV	292	19
nonsynonymous SNV	293	17
nonsynonymous SNV	1220	756

nonsynonymous SNV	1724	1142
nonsynonymous SNV	268	18
nonsynonymous SNV	424	26
nonsynonymous SNV	198	10
nonsynonymous SNV	180	9
nonsynonymous SNV	196	10
nonsynonymous SNV	196	10
nonsynonymous SNV	174	9
nonsynonymous SNV	175	9
nonsynonymous SNV	476	26
nonsynonymous SNV	267	16
nonsynonymous SNV	169	11
nonsynonymous SNV	156	10
nonsynonymous SNV	1221	172
frameshift deletion	1065	540
nonsynonymous SNV	1008	439
nonsynonymous SNV	226	14
nonsynonymous SNV	184	14
nonsynonymous SNV	356	32
nonsynonymous SNV	320	66
nonsynonymous SNV	247	43
nonsynonymous SNV	240	13
nonsynonymous SNV	157	8
nonsynonymous SNV	466	190
nonsynonymous SNV	610	216
nonsynonymous SNV	181	10
nonsynonymous SNV	242	20
nonsynonymous SNV	200	17
nonsynonymous SNV	150	8
nonsynonymous SNV	650	52
nonsynonymous SNV	1958	458
nonsynonymous SNV	2000	572
nonsynonymous SNV	589	77
nonsynonymous SNV	164	20
stopgain	203	12
nonsynonymous SNV	294	22
nonsynonymous SNV	218	12
.	177	12
nonsynonymous SNV	423	23
nonsynonymous SNV	981	260
nonsynonymous SNV	199	12

nonsynonymous SNV	681	136
nonsynonymous SNV	198	10
nonsynonymous SNV	2114	527
nonsynonymous SNV	283	17
nonsynonymous SNV	1779	155
nonsynonymous SNV	157	10
nonsynonymous SNV	198	35
stopgain	1028	286
nonsynonymous SNV	2013	547
stopgain	327	20
nonsynonymous SNV	4174	1572
nonsynonymous SNV	177	23
nonsynonymous SNV	157	12
nonsynonymous SNV	2037	415
nonsynonymous SNV	165	11
nonsynonymous SNV	1649	361
nonsynonymous SNV	386	20
nonframeshift deletion	827	365
nonsynonymous SNV	567	33
nonsynonymous SNV	173	10
nonsynonymous SNV	2627	1003
nonsynonymous SNV	2692	1357
nonsynonymous SNV	213	13
nonsynonymous SNV	188	13
nonsynonymous SNV	199	16
nonsynonymous SNV	241	23
nonsynonymous SNV	159	9
nonsynonymous SNV	465	28
nonsynonymous SNV	1345	601
nonsynonymous SNV	492	27
nonsynonymous SNV	1516	710
nonsynonymous SNV	193	10
stopgain	1365	635
nonsynonymous SNV	399	183
nonsynonymous SNV	1557	708
nonsynonymous SNV	1553	495
nonsynonymous SNV	153	8
nonsynonymous SNV	204	11
nonsynonymous SNV	1785	219
nonsynonymous SNV	1517	103
nonsynonymous SNV	711	391

nonframeshift deletion	1084	812
nonsynonymous SNV	265	135
stopgain	261	152
nonsynonymous SNV	156	9
nonsynonymous SNV	1233	441
.	189	10
nonsynonymous SNV	184	18
nonsynonymous SNV	218	14
nonsynonymous SNV	151	14
nonsynonymous SNV	174	10
nonsynonymous SNV	395	21
nonsynonymous SNV	1687	675
nonsynonymous SNV	2076	117
nonsynonymous SNV	247	21
nonsynonymous SNV	1339	1066
nonsynonymous SNV	2058	966
nonsynonymous SNV	2086	1017
nonsynonymous SNV	205	12
nonsynonymous SNV	376	20
nonsynonymous SNV	222	27
nonsynonymous SNV	277	65
nonsynonymous SNV	445	213
nonsynonymous SNV	375	66
nonsynonymous SNV	195	10
nonsynonymous SNV	175	9
nonsynonymous SNV	171	17
nonsynonymous SNV	164	10
.	1102	508
nonsynonymous SNV	1153	419
nonsynonymous SNV	170	26
nonsynonymous SNV	458	224
nonsynonymous SNV	242	17
nonsynonymous SNV	289	16
nonsynonymous SNV	156	12
nonsynonymous SNV	189	10
nonsynonymous SNV	198	56
nonsynonymous SNV	304	44
nonsynonymous SNV	167	10
nonsynonymous SNV	159	11
nonsynonymous SNV	1052	85
.	156	8

.	748	368
frameshift deletion	2208	1005
nonsynonymous SNV	975	347
nonsynonymous SNV	199	15
nonsynonymous SNV	190	19
stopgain	377	19
nonsynonymous SNV	411	21
stopgain	524	28
nonsynonymous SNV	952	221
.	320	16
.	177	14
nonsynonymous SNV	1461	126
nonsynonymous SNV	422	154
nonsynonymous SNV	199	10
nonsynonymous SNV	353	19
nonsynonymous SNV	1489	340
nonsynonymous SNV	319	18
nonsynonymous SNV	903	249
nonsynonymous SNV	191	14
stopgain	397	70
nonsynonymous SNV	368	108
stopgain	159	10
nonsynonymous SNV	335	18
stopgain	359	80
nonsynonymous SNV	325	18
nonsynonymous SNV	164	12
nonsynonymous SNV	160	10
nonsynonymous SNV	348	20
nonsynonymous SNV	162	9
nonsynonymous SNV	279	18
nonsynonymous SNV	2058	222
nonsynonymous SNV	915	205
nonsynonymous SNV	421	25
nonsynonymous SNV	448	71
nonsynonymous SNV	259	16
nonsynonymous SNV	323	168
nonsynonymous SNV	1272	189
nonsynonymous SNV	2755	377
nonsynonymous SNV	588	137
nonsynonymous SNV	2317	658
nonsynonymous SNV	152	11

nonsynonymous SNV	266	20
nonsynonymous SNV	1055	204
nonsynonymous SNV	981	342
nonsynonymous SNV	851	44
nonsynonymous SNV	774	53
stopgain	838	105
nonsynonymous SNV	299	33
nonsynonymous SNV	304	17
nonsynonymous SNV	316	17
nonsynonymous SNV	1381	120
nonsynonymous SNV	602	57
nonsynonymous SNV	2598	749
nonsynonymous SNV	209	15
stopgain	2253	1051
nonsynonymous SNV	333	20
nonsynonymous SNV	158	8

Variant Allele Fraction
0.74375
0.6043956
0.25477707
0.88418998
0.62109375
0.26925013
0.35551142
0.7834736
0.44868735
0.27666399
0.35753053
0.17241379
0.62566845
0.49352179
0.80716723
0.25890736
0.69176471
0.40355125
0.82325581
0.64907652
0.67193676
0.61935484
0.05921053
0.19312796
0.06578947
0.50154321
0.06859504
0.75144509
0.22065728
0.37562189
0.5952381
0.69966997
0.56962025
0.32352941
0.55965909
0.26065891
0.42412194
0.31147541
0.48596939
0.05263158

0.26451613
0.35939643
0.49710025
0.05263158
0.0513834
0.05194805
0.06280193
0.06145251
0.09836066
0.26835443
0.05524345
0.06153846
0.43971631
0.47420635
0.26123876
0.05027933
0.05454545
0.05521472
0.05853659
0.30618337
0.13356766
0.47978227
0.19134078
0.2288
0.06622517
0.09008097
0.06060606
0.46896955
0.05102041
0.08383233
0.1146789
0.06606607
0.06481481
0.06914894
0.17843866
0.4496337
0.10161361
0.19811321
0.48631124
0.05204461
0.06763285

0.86760281
0.18378378
0.05159705
0.41632231
0.05555556
0.05681818
0.07070707
0.07594937
0.05590062
0.06405694
0.3040293
0.28169014
0.82515823
0.05309734
0.05844156
0.49504951
0.09022556
0.06329114
0.07453416
0.63970588
0.07365439
0.44708348
0.37330929
0.22945205
0.38211382
0.40303907
0.13459621
0.38772788
0.07647059
0.38845144
0.0516129
0.06622517
0.23891892
0.49074074
0.13693694
0.11824324
0.12429379
0.14040115
0.06349206
0.05555556
0.07096774

0.06632653
0.14347826
0.05555556
0.68468469
0.05464481
0.07783019
0.0625
0.05714286
0.06355932
0.06862745
0.85785632
0.05714286
0.39448622
0.42160279
0.4602961
0.06481481
0.0610687
0.20389884
0.16666667
0.11328125
0.77300614
0.05298013
0.07191781
0.06214689
0.07535795
0.55393401
0.1496063
0.145
0.16247672
0.50655022
0.05250597
0.12107789
0.47918512
0.38964578
0.05095541
0.05319149
0.05357143
0.08672566
0.05072464
0.08737864
0.87825182

0.3463035
0.21205357
0.24675325
0.1210856
0.15368852
0.14178674
0.05164319
0.11875
0.06222222
0.24110032
0.50487106
0.41409435
0.18820225
0.61111111
0.46285085
0.25369004
0.51288772
0.05305822
0.05405405
0.05660377
0.14583333
0.07429719
0.52369668
0.51757812
0.1075
0.07142857
0.05247813
0.37168142
0.61728395
0.48224299
0.06172839
0.05882353
0.14215686
0.12101911
0.0656168
0.26663147
0.05084746
0.08910891
0.06506849
0.05802048
0.61967213

0.66241299
0.06716418
0.06132075
0.05050505
0.05
0.05102041
0.05102041
0.05172414
0.05142857
0.05462185
0.05992509
0.06508876
0.06410256
0.14086814
0.50704225
0.43551587
0.0619469
0.07608696
0.08988764
0.20625
0.17408907
0.05416667
0.05095541
0.40772532
0.35409836
0.05524862
0.08264463
0.085
0.05333333
0.08
0.23391216
0.286
0.13073005
0.12195122
0.0591133
0.07482993
0.05504587
0.06779661
0.05437352
0.26503568
0.06030151

0.19970631
0.05050505
0.24929044
0.06007067
0.0871276
0.06369427
0.17676768
0.27821012
0.27173373
0.06116208
0.37661715
0.1299435
0.07643312
0.20373098
0.06666667
0.21892056
0.05181347
0.44135429
0.05820106
0.05780347
0.38180434
0.50408618
0.06103286
0.06914894
0.08040201
0.09543569
0.05660377
0.06021505
0.44684015
0.05487805
0.46833773
0.05181347
0.46520146
0.45864662
0.45472062
0.31873793
0.05228758
0.05392157
0.12268908
0.06789716
0.54992968

0.74907749
0.50943396
0.58237548
0.05769231
0.35766423
0.05291005
0.09782609
0.06422018
0.09271523
0.05747126
0.05316456
0.40011855
0.05635838
0.08502024
0.7961165
0.46938776
0.48753595
0.05853659
0.05319149
0.12162162
0.23465704
0.47865168
0.176
0.05128205
0.05142857
0.09941521
0.06097561
0.46098004
0.36339983
0.15294118
0.48908297
0.07024793
0.05536332
0.07692308
0.05291005
0.28282828
0.14473684
0.05988024
0.06918239
0.08079848
0.05128205

0.49197861
0.45516304
0.35589744
0.07537688
0.1
0.05039788
0.05109489
0.05343511
0.23214286
0.05
0.07909605
0.0862423
0.36492891
0.05025126
0.05382436
0.22834117
0.05642633
0.27574751
0.07329843
0.17632242
0.29347826
0.06289308
0.05373134
0.22284123
0.05538461
0.07317073
0.0625
0.05747126
0.05555556
0.06451613
0.10787172
0.22404372
0.05938242
0.15848214
0.06177606
0.52012384
0.14858491
0.1368421
0.2329932
0.28398791
0.07236842

0.07518797
0.19336493
0.34862385
0.05170388
0.06847545
0.12529833
0.11036789
0.05592105
0.05379747
0.08689355
0.09468438
0.28829869
0.07177034
0.46648913
0.06006006
0.05063291
